# Nutrient Diagnosis of *Eucalyptus* at the Factor-Specific Level Using Machine Learning and Compositional Methods

**DOI:** 10.3390/plants9081049

**Published:** 2020-08-18

**Authors:** Betania Vahl de Paula, Wagner Squizani Arruda, Léon Etienne Parent, Elias Frank de Araujo, Gustavo Brunetto

**Affiliations:** 1 Departemento dos Solos, Universidade Federal de Santa Maria, Av. Roraima, 1000-Camobi, Santa Maria-RS 97105-900, Brazil; wagnersquizani@hotmail.com (W.S.A.); leon-etienne.parent@fsaa.ulaval.ca (L.E.P.); brunetto.gustavo@gmail.com (G.B.); 2 Department of Soils and Agrifood Engineering, Laval University, Quebec, QC G1V 0A6, Canada; 3 Soil and Management Researcher of CMPC-Cellulose Rio Grandense, Rua São Geraldo 1680-Guaíba–RS, Brazil; elias.araujo@cmpcrs.com.br

**Keywords:** compatibility intervals, Euclidean distance, Humboldtian loci, centered log ratios, machine learning

## Abstract

Brazil is home to 30% of the world’s *Eucalyptus* trees. The seedlings are fertilized at plantation to support biomass production until canopy closure. Thereafter, fertilization is guided by state standards that may not apply at the local scale where myriads of growth factors interact. Our objective was to customize the nutrient diagnosis of young *Eucalyptus* trees down to factor-specific levels. We collected 1861 observations across eight clones, 48 soil types, and 148 locations in southern Brazil. Cutoff diameter between low- and high-yielding specimens at breast height was set at 4.3 cm. The random forest classification model returned a relatively uninformative area under the curve (AUC) of 0.63 using tissue compositions only, and an informative AUC of 0.78 after adding local features. Compared to nutrient levels from quartile compatibility intervals of nutritionally balanced specimens at high-yield level, state guidelines appeared to be too high for Mg, B, Mn, and Fe and too low for Cu and Zn. Moreover, diagnosis using concentration ranges collapsed in the multivariate Euclidean hyper-space by denying nutrient interactions. Factor-specific diagnosis detected nutrient imbalance by computing the Euclidean distance between centered log-ratio transformed compositions of defective and successful neighbors at a local scale. Downscaling regional nutrient standards may thus fail to account for factor interactions at a local scale. Documenting factors at a local scale requires large datasets through close collaboration between stakeholders.

## 1. Introduction

*Eucalyptus* plantations cover 20 × 10^6^ ha worldwide to provide raw material for wood, paper, biofuel, firewood, and charcoal [1]. Brazil is the world leader, producing *Eucalyptus* on 6 × 10^6^ ha with an average yield of 36 m^3^ ha^−1^ year^−1^ [2]. While *Eucalyptus* is adapted to low-fertility soils, nutrient supply, especially N and K [3,4,5,6] can limit stand productivity [1,7,8]. Fertilization was found to increase wood production of *Eucalyptus grandis* by 28% and irrigation by another 30% to reach potential outcome of 83 m^3^ ha^−1^ year^−1^, where the most yield-impacting factors are set at near optimum levels [9].

*Eucalyptus* seedlings are heavily fertilized at planting to prevent nutrient deficiency and non-uniform tree growth until canopy closure [8]. Thereafter, fertilization aims to recharge the soil–plant system with nutrients where initial inputs appeared ineffective. Fertilization decisions are usually taken based on soil and tissue tests. Plant tissue tests are thought to integrate the effects of growth factors on crop performance [10]. Regional tissue standards [11] have been developed to guide fertilization of *Eucalyptus* seedlings [8,12,13] and of trees more than 6 years of age [14]. No standards have been developed for trees of intermediate age.

Tissue tests are generally interpreted using general nutrient concentration ranges or nutrient ratios. First, the statistical treatment of concentration values to generate intervals may lead to biased or wrong results [15]. In addition, the concept of statistically-derived ranges has been recently challenged by a concept of “compatibility intervals” to avoid taking wrong dichotomous decisions on rejection [16]. Furthermore, regional nutrient ratios or product standards and expressions have been elaborated based on heroic assumptions such as universality and timeless nutrient norms, and function additivity [17].

The suitability of downscaling regional standards for application at a local scale where myriads of factor interactions occur has been minimally addressed [18]. Errors on interactions [19] that involve environmental factors, genetics, nutrients and time may reduce diagnostic efficiency because factor effects are averaged across factors at a regional scale. Several factors can affect plant growth [20]. Soil type, climatic conditions [11], clone nutrient-use efficiency [21,22], and management factors such as stand quality, tree spacing, fertilization, and even tree pruning and thinning [8] vary widely, leading to contrasting fertilizer recommendations for *Eucalyptus* stands [9]. Nevertheless, data sets must be well documented to customize nutrient diagnosis at the specified combination of factors.

Humboldtian principles of quantitative biogeography require integrating data collected in living systems [23]. Humboldtian patterns can be extracted using methods of artificial intelligence to solve complex problems that are beyond human capabilities [24,25]. A heuristically simple factor-specific diagnostic approach is to compare defective and successful Humboldtian loci across a set of features using compositional and classification or regression machine learning (ML) methods [18,26,27]. In such a case, the assumption that factors other than the ones being addressed are equal or at optimum levels [28] is replaced by the assumption that documented factors other than the ones being addressed are comparable. Only non-documented factors must be assumed to be equal.

Compositional data are strictly positive data with constraints such as closure to measurement unit or scale, missing values, data censoring, ethical data collection, data merging, levelling of different datasets from various sources, sample design [29], accuracy of measurements, and handling of zeroes [30]. To handle numerical constraints, compositional data should be log-ratio transformed before conducting statistical analyses [15,31]. Machine learning methods can also unravel complex patterns in data [24,25]. Machine learning (ML) and compositional data analysis (CoDa) methods thus provide unprecedented tools to conduct factor-specific nutrient diagnosis and verify the relevance of downscaling regional standards at a local scale.

We hypothesized that (1) the productivity of *Eucalyptus* following plantation depends on tissue composition and local features, (2) log-ratio transformations increase the accuracy of ML models, and (3) regional diagnosis can be downscaled reliably to factor-specific levels. Our objective was to customize tissue nutrient diagnosis of young *Eucalyptus* trees at a local scale.

## 2. Materials and Methods

### 2.1. Data Set

The data set comprised 1861 observations on young *Eucalyptus* trees across eight clones, 48 soil types (with predominance of types Typic Hapludalf and Udorthent), and 148 locations in southern Brazil. Most trees (97%) were between 0.9 and 1.1 years old following plantation. The clones were *Eucalyptus* spp. (*E. benthamii, E. saligna, E. dunnii, E. urophylla, E. urophylla S. T. Blake, E. urophylla × E. globulus, E. urophylla × E. grandis, E. urophylla Blake × E. grandis Hill, E. camaldulensis × E. grandis × E. urophylla*) collected on the Coastal Region of Rio Grande do Sul, Southern Brazil. Tree seedlings had been grown in 100 mL containers for 5–6 months to reach a root collar diameter of 3–4 mm and plant height of 30–40 cm before planting [8]. Tree spacing was 3 m by 3 m for an average plantation density of 1100 trees ha^−1^.

The regional climate is humid temperate subtropical according to the international Köppen-Geiger classification. Winters are moderately cold with frost. Summers are hot with day temperatures most often >30 °C. Rainfall is well distributed throughout the year, with annual accumulations ranging from 1000 mm to >2000 mm [32]. The soil classification was coded at each site according to the Brazilian soil classification system [33]. The data set did not include pest management, pest damage, and meteorology.

### 2.2. Fertilization

Soil tests were not available, but fertilization followed regional guidelines [11]. At plantation, fertilizers were manually applied in planting holes or grooves, or besides tree seedlings, and then mixed with soil. Fertilization rates varied between 15 and 45 kg N ha^−1^ or more depending on soil organic matter content. The P (0–57 kg P ha^−1^) and K (0–108 kg K ha^−1^) fertilization depended on soil P and K tests, respectively. Thereafter, P and K fertilizers were applied at rates of up to 22 kg P ha^−1^ and up to 40 kg K ha^−1^, respectively, based on regional tissue nutrient standards. Additional N supply of 15–45 kg N ha^−1^ depended on soil organic matter content and wood marginal yield exceeding 40 m^3^ ha^−1^ year^−1^. Micronutrient levels could have been impacted by applications of composts, fertilization, fungicides, and lime. Micronutrients were applied as needed at rates of 1 kg B ha^−1,^ 1.5 kg Zn ha^−1^, and 1 kg Cu ha^−1^.

### 2.3. Plant Measurements and Analysis

Yearly, between January and March, plant height was measured using a metric tape. Stem diameter was measured as diameter at breast height (DBH ≈1.3 m in height). Plant height and tree diameter are closely related to the wood volume of *Eucalyptus* [9]. The DBH was thus used as a target variable to run ML models.

Yearly, from February to April, leaves were collected in the middle tier of the annual growth (4th to 5th leaf from branch tip) from at least ten trees per site. Eleven nutrients were analyzed [34]. Foliar N was quantified by micro-Kjeldahl. The S, P, K, Ca, Mg, Zn, Cu, Mn, Fe, and B foliar concentrations were determined by ICP-OES after digestion in a mixture of nitric and perchloric acids.

### 2.4. Log-Ratio Transformation Techniques

Before the work of Aitchison [15], compositions were addressed using concentrations or pairwise ratios between components $x_{i}$ and $x_{j}$ and expressed as $x_{i}/x_{j}\;$ [35]. Pairwise ratios required (1) selecting $x_{i}/x_{j}$ or its inverse $x_{j}/x_{i}$ based on variance ratios between low-yielding and high-yielding subpopulations, (2) reflective equations, and (3) assumptions on additivity to compute functions and indices [17]. While the logarithmic scale avoids large numbers of decimals [36], log-transformed pairwise ratios allows recovering reflectivity, i.e., $ln\left(x_{i}/x_{j}\;\right)=-ln\left(x_{j}/x_{i}\right)$. There are D×(D−1)/2 pairwise log ratios (pwlr) derived from D concentration data that generate redundant information in multivariate models.

The pwlr computed as $ln\left(x_{i}/x_{j}\right)$ is also called a log contrast, i.e., $ln\left(x_{i}/x_{j}\right)=ln\left(x_{i}\right)-ln\left(x_{j}\right)$. The composition is closed to some total by computing a filling value (Fv) between the total and the sum of quantified components. The pwlr values for a given nutrient can be compressed into a single centered log ratio (*clr*) [26], as follows for tissue N:

(1) $$\begin{array}{c} clr_{N}=ln\left(\frac{N}{G}\right)=ln\left(\sqrt[{11}]{\frac{N}{N}\times \frac{N}{P}\times \frac{N}{K}\times \frac{N}{Mg}\times \frac{N}{Ca}\times \frac{N}{B}\times \frac{N}{Cu}\times \frac{N}{Zn}\times \frac{N}{Mn}\times \frac{N}{Fe}\times \frac{N}{Fv}}\right)=\\ \frac{1}{11}\times \left[ln\left(\frac{N}{N}\right)+ln\left(\frac{N}{P}\right)+\ldots ln\left(\frac{N}{Fv}\right)\right]=\frac{1}{11}\left[ln\left(\frac{N}{P}\right)+\ldots ln\left(\frac{N}{Fv}\right)\right] \end{array}$$

where *N* is the tissue nitrogen concentration, and G is the geometric mean across components (including the nutrient itself and the filling value), all expressed using the same measurement unit or scale. The computation of *G* does not accept missing data unless imputed or approximated from detection limits [30]. The clr transformation provides a solid mathematical ground for the integration of dual ratios [37] and avoids assumptions on additivity and reflectivity as required for Diagnosis and Recommendation Integrated System (DRIS) computations [38]. The clr transformation can account for all dual nutrient interactions and therefore reduces the inter-relationships among nutrients compared to raw concentrations as shown by the Kaiser-Meyer-Olkin (KMO) measure of sampling adequacy in principal component analysis [39].

The D-part compositions can be compressed into D-1 isometric log ratios or orthonormal balances [31], the exact number of degrees of freedom available in compositions [40]. The orthonormal balances between selected subsets of components at the numerator and denominator are computed as follows:

(2) $$ilr_{i}=\sqrt{\frac{rs}{r+s}}ln\left(\frac{G_{N}}{G_{D}}\right)$$

where *r* and *s* are numbers of components at the numerator and denominator, respectively, and $G_{N}$ and $G_{D}$ are geometric means of components at the numerator and denominator, respectively.

Orthogonality is a concept of linear independence [41]. The *ilr* transformation is the most appropriate log ratio transformation technique to conduct multivariate analysis of compositional data, avoiding spurious correlations and singular matrix [42]. While orthonormal balances can be arranged into meaningful combinations in line with the objectives of the study [43], multivariate distances and the results of multivariate analysis remain the same whatever the arrangement of components into balances, due to orthogonality between *ilr* variables.

### 2.5. Regional Diagnosis

The *clr* indices are computed from mean and standard deviation of *clr* values for the nutritionally balanced subpopulation as follows [37]:

(3) $$I_{i}=\frac{\left(clr_{i}-clr_{i}^{*}\right)}{SD_{i}^{*}}$$

where $I_{i}$ is the *clr* index of nutrient i, $clr_{i}$ is the *clr* value for the diagnosed specimen, and $clr_{i}^{*}$ and $SD_{i}^{*}$ are the mean and standard deviation of nutrient *i* used as references. Nutrient indices are ranked in the order of their limitation to yield from the most negative to the most positive *clr* index. To assign a probability level to D-parts compositions, Compositional Nutrient Diagnosis (CND) indices may be added up to a squared multivariate distance distributed like a proximate $\chi^{2}$ variable with D−1 degrees of freedom [44].

### 2.6. Local Diagnosis

To conduct nutrient diagnoses at a factor-specific level, the Euclidean distance ϵ between two D-part compositions can be computed across *clr* values as follows [26]:

(4) $$\epsilon =\sqrt{\sum_{i=1}^{D}{\left(clr_{i}-clr_{i}^{*}\right)}^{2}}=\sqrt{\sum_{i=1}^{D}{\left(ilr_{i}-ilr_{i}^{*}\right)}^{2}}$$

where $clr_{i}$ and $clr_{i}^{*}$ or $ilr_{i}$ and $ilr_{i}^{*}$ represent high yield and nutrient balance TN compositions, respectively. Successful TN specimens are productive specimens showing a small Euclidean distance from the diagnosed specimen. Because $\overset{D}{\underset{i=1}{\sum }}clr_{i}=0$, nutrients can be ranked in the numerical order of *clr* differences from the most negative (relative shortage) to the most positive (relative excess).

### 2.7. Statistical Analysis

The *clr* biplot was drawn using the freeware Codapack 2.02.21 (http://ima.udg.edu/codapack/) to document the relative contribution of nutrient concentrations to tissue compositions. The ML classification models were run using the freeware Orange vs. 3.24 (Bioinformatics Lab, Ljubljana, Slovenia) by relating crop yield (target variable) to growth-impacting features. Overfitting due to too many features could be handled by ML models [45]. Nevertheless, this is a key issue in ML because the size and number of features differ between concentration, pairwise log ratio (pwlr), centered log ratio (clr) and isometric log ratio (ilr) expressions and this may impact the model accuracy [35].

The *Eucalyptus* population was partitioned into low- and high-yielding subpopulations based on a critical DBH of 4.3 cm as an economically viable yield target. The random forest (RF), neural network (NN), naïve Bayes, support vector machine (SVM), KNN, Adaboost, and stochastic gradient decent (SGD) models were tested in cross-validation. The results of ten successive runs were averaged after randomly removing 10% of the data. Model accuracy was assessed by area under the curve (AUC). An AUC between 0.7 and 0.9 is informative [46]. The contribution of features to model accuracy can be assessed by removing one feature at the time. The confusion matrix of the machine learning model classified specimens into four quadrants as follows [47]:

True negative specimens (TN): high productivity and adequate nutrient balance (negative response to fertilization). They are located in the upper left quadrant of the confusion matrix.

False negative specimens (FN): low productivity despite adequate nutritional balance (negative response to fertilization, some other factor limiting yield). They are located in the lower left quadrant of the confusion matrix.

False positive specimens (FP): high productivity despite nutrient imbalance (contamination, sub-optimal concentration, excess or luxury consumption of some nutrient). They are located in the upper right quadrant of the confusion matrix.

True positive specimens (TP): low productivity and nutritional imbalance (positive response to fertilization). They are located in the lower right quadrant of the confusion matrix.

Classification accuracy (CA) was computed as follows [44]:

(5) $$CA=\frac{TN+TP}{TN+FN+TP+FP}$$

Data partitioning followed principles of data interpretation similar to those used for the human response to drugs in clinical biology [46]. Data partitioning in the confusion matrix avoids merging balanced and imbalanced specimens at high yield level as in DRIS [17,38]. Nutrient imbalance of high yielding specimens is due to over-fertilization leading to luxury consumption of nutrients that should be avoided, or to nutrient contamination that unduly increases the variation of nutrient levels and could bias nutrient diagnosis. Nutrient compatibility intervals [16] at a high yield level were computed from TN quartiles. While FN specimens are also nutritionally balanced and could be considered to compute reference values at a regional scale, they do not provide realistic yield targets as shown in the data set at a local scale. Successful TN specimens are local references to correct defective compositions at the specified combination of factors.

## 3. Results

### 3.1. Descriptive Statistics and Exploratory Analyses

There was a large variation in tissue compositions (Table 1).

**Figure 1:**
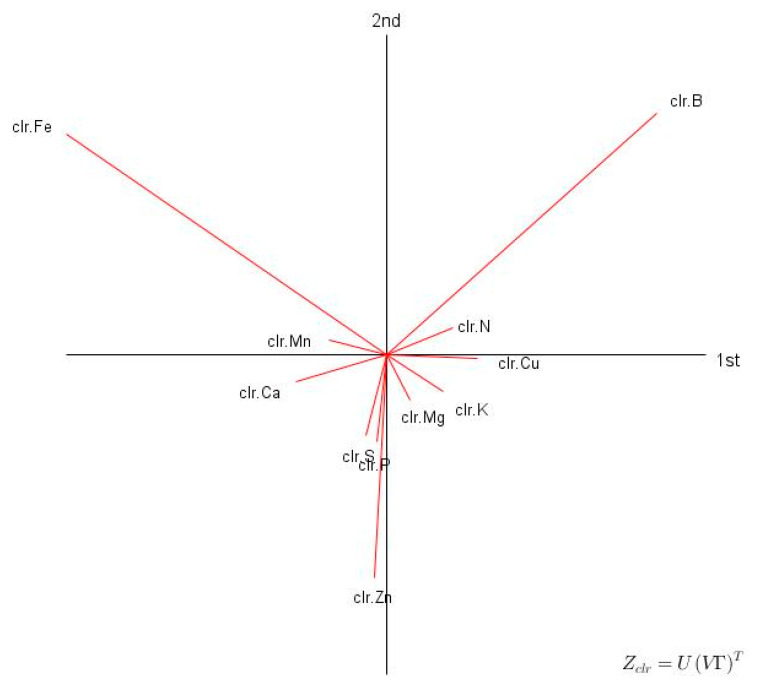
Clr biplot (CoDapack v2.02.21) of tissue compositions of 1861 young *Eucalyptus* trees in southern Brazil.

The *clr* biplot showed that Zn, B and Fe contributed the most to total variance of *Eucalyptus* tissue compositions (Figure 1), indicating wide variation in soil genesis (Fe) and management decisions such as applications of fungicides and organic residues. The large variation in Zn and B levels may have been impacted by composts, fertilization, fungicides, and liming.

### 3.2. Machine Learning Models

The RF, naïve Bayes and NN models were found to be informative (Table 2). Adaboost, SVM, KNN and SGD were not informative (AUC < 0.7).

The RF model was preferred to the naïve Bayes model to avoid assumptions on feature independence in interactive Humboldtian living systems such as *Eucalyptus* ecosystems. While the RF model can deal with over-fitting of partition trees, it may be affected by the choice of the expression [35]. The raw concentration and *clr* expressions returned the highest accuracies (Table 3). The raw concentration expression is preferable because the model is not affected by missing or zero values that impair computing log ratios.

### 3.3. Nutrient Intervals at a Regional Scale

Regional nutrient standards can be assessed from TN quartiles, which are specimens showing high yield and adequate nutrient balance. For this reason, TN specimens are considered the reference compositions for diagnostic purposes at a local scale. Where the number of TN specimens is too small, FN specimens could also be considered at a regional scale. Among the 529 TN specimens, 40 were outside the target age range of 0.9–1.1-years-old and were thus discarded, leaving 489 TN specimens to compute the TN quartile compatibility intervals concentration (Table 4).

The present Brazilian standards for *Eucalyptus* [11] overlapped the across-factor quartile ranges of the TN specimens for N, P, K, and Ca, and but were out of range for Mg and micronutrients. As shown in Table 5, state standards and TN quartiles returned similar diagnoses 12 times out of 22 attempts, indicating a high risk of wrong fertilization decisions. The decision to implement corrective measures is thus influenced by the choice of specific boundaries for compatibility intervals. Machine learning model prediction and compositional analysis tools can avoid diagnosing nutrient levels using fixed compatibility intervals.

### 3.4. Regional vs. Local Diagnosis

Regional diagnosis is conducted by computing *clr* indices from *clr* means and standard deviations of TN specimens, assuming that factors other than the nutritional ones are equal or at near-optimum levels at a regional scale (Table 6). At a local scale, uncontrollable factors (e.g., soil profile) or ones that are difficult to control (e.g., P, Cu, Zn and Fe accumulation in soil) could be accounted for by site analogy. The ML prediction model compares factor-specific defective compositions to the closest TN specimens sharing the same features. The criterion for closeness between compositions is the Euclidean distance at the specified combination of factors. We selected ten close TN specimens to conduct nutrient diagnosis at the specified combination of factors.

Factor analogy between defective and successful specimens at the specified combination of factors is assessed in the TN data set to diagnose nutrient problems in defective specimens. At the clone × age interaction level, AUC of the RF model was 0.71 and the model was still informative. The RF model predicted that the probabilities for the diagnosed specimens in Table 5 to be classified as high yielders were 48% and 36% at sites #1 and #2, respectively, indicating a need for corrective measures.

**Figure 2:**
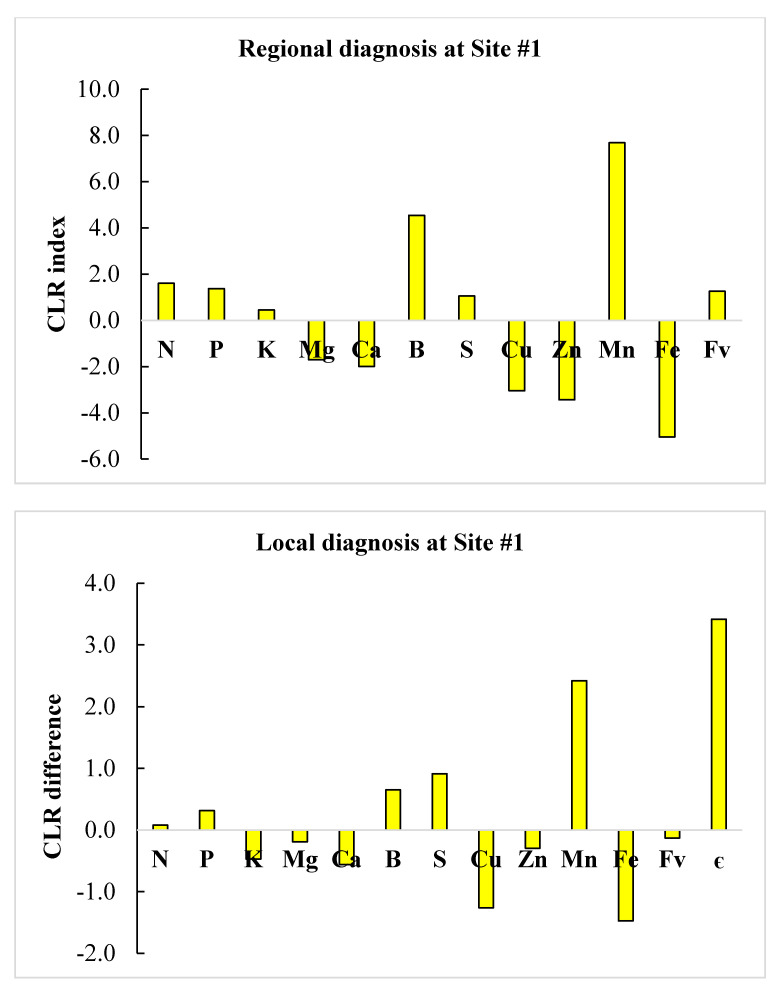
Comparison between regional (**top**) and local (**bottom**) nutrient diagnoses at Site #1 using centered log ratios (CLR) of regional TN standards or a successful local neighbor as measured by the Euclidian distance (є).

**Figure 3:**
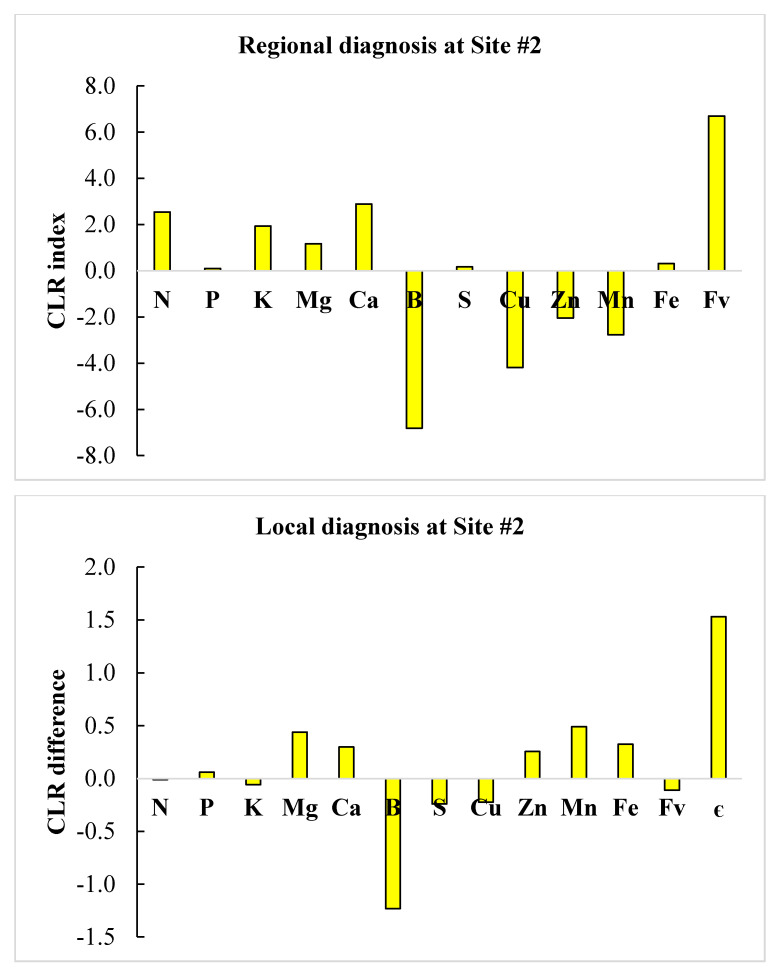
Comparison between regional (**top**) and local (**bottom**) nutrient diagnoses at Site #2 using centered log ratios (CLR) of regional TN standards or a successful local neighbor as measured by the Euclidian distance (є).

We selected successful neighbors showing DBH > 5 cm at the clone × age interaction level and where nutrient requirements were most parsimonious to minimize cost of adjusting nutrient management. Regional diagnosis using *clr* standards in Table 5 and local diagnosis of the two defective compositions are illustrated in the form of histograms in Figure 2 and Figure 3. Close successful neighbors reached a DBH of 5.43–5.44 cm compared to 4.06 cm and 1.71 at sites #1 and #2, respectively, indicating high potential to boost plant growth using appropriate corrective measures.

At site #1, there was relative Mn excess at both regional and local scales. At the regional scale, B and N ranked second and third in relative excess, while Fe, Zn and Cu ranked in a descending order of relative nutrient shortage. At the local scale, S and B ranked second and third in relative excess, while Fe and Cu ranked in a descending order of relative nutrient shortage. As a result, Fe, Zn and Cu should be added following regional diagnosis at site #1, while only Fe and Cu would be required following local diagnosis. At site #2, B appeared to be in relative shortage at both regional and local scales. At the regional scale, N and Ca showed relative excess, while B and Cu ranked in a descending order of relative nutrient shortage. At the local scale, only B appeared to limit yield. As a result, B and Cu should be added and N reduced following regional diagnosis at site #2, while only B would be required according to local diagnosis.

## 4. Discussion

### 4.1. ML Model

The AUC of the RF model that included features available in the data set was 0.78, indicating that the model was informative. The accuracy of the RF classification model was 0.72 compared to more than 0.80 for most tested crops [48]. Raw concentrations with no need to impute missing values returned higher model accuracy than log ratios. On the other hand, zero or missing values make it impossible to compute log ratios, potentially reducing the size of the data set available to run ML models if imputation is not possible or there are too many zeroes in the data set.

Compared to compositional models that report nutrient interactions as ratios or multi-ratios, ML models address factor interactions as combinations of factors at a given geographical scale. This is different from the definition of factor interactions in statistical models. Errors on interactions occur when comparing means of main effects where interactions were significant or reporting means at the interaction level where the interactions were not significant [19]. While ecological patterns result from myriads of interactive processes, most statistical models can solve only a limited number of interactions between factors [49]. In ML models, the concept of significance is replaced by an assessment of increased accuracy after adding potentially contributing factors whatever their size effect or significance. The minimum number of combined factors to reach high model accuracy is the minimum data set required to solve the problem under study with smallest effort on data collection.

In statistical analysis, claiming ‘statistically non-significant’ differences does not mean that there was no difference at all, leading to potential conflictual conclusions [16]. Confidence intervals should thus be renamed “compatibility intervals” to embrace uncertainty on interpretation. In comparison, ML methods include growth-impacting factors, avoiding the accept/reject “dichotomania” of either adding or removing features based on significance to assess factor contribution to model accuracy.

Critical concentration ranges bear different meanings. They can be presented as statistically derived intervals such as boxplots and confidence intervals, or as physiological response patterns to nutrient additions where critical boundaries are defined arbitrarily at 90–95% maximum yield. Boxplots are easily derived from regional crop surveys where nutrient treatments are not varied systematically, by assigning tissue nutrient compositions to yield classes. Tissue nutrient thresholds require varying doses in one-nutrient or factorial experiments, but such trials are site-specific and expensive. In both cases, concentration ranges are fixed values leading dichotomous decisions. Claiming that some nutrients of the diagnosed specimen fall outside the “critical concentration range” does not mean that the specimen is nutritionally imbalanced. It merely reflects some incompatibility between diagnosed concentrations and the statistically or physiologically derived concentration ranges.

It appears nonsensical that 50% of the TN specimens in the present study would fall outside the boundaries delineated by boxplots for diagnostic purposes. It is even more surprising to find just one TN specimen surviving after diagnosing the whole TN data set across all compatibility intervals in Table 4, an insignificant success rate (one out of 489 observations!). Regional compatibility intervals also proved to be a complete failure (zero success). Indeed, current critical nutrient ranges are assemblages of separately derived concentration ranges pasted together to generate a “Frankenstein-built” diagnostic tool that denies nutrient interactions. Indeed, assuming normal data distribution within normalized critical ranges, it can be shown geometrically that diagnosing by nutrient compatibility intervals collapses in the Euclidean hyper-space as more nutrients are being diagnosed to fully capture nutrient imbalance [50]. While there is a false belief that crossing the threshold of statistical significance is sufficient as a proof [16], it is similarly a false belief that crossing critical concentration ranges is enough to demonstrate nutrient imbalance. This is why critical nutrient concentration ranges (compatibility intervals) should be abandoned for diagnostic purposes as strongly impacted by errors on nutrient interactions.

While nutrients interact with each other, *clr* or *ilr* variables can project them into the Euclidean hyper-space of plant nutrients to avoid disastrous conclusions. The compositional methods view nutrient compositions as entities, i.e., unique combinations of nutrients in a tissue. Nutrients interact between them in several ways [17,51], and this can be handled by log ratio transformations [37]. The distance between two equal-length compositions is computed as a Euclidean distance using *clr* or *ilr* variables. The *clr* differences can rank nutrients in the order of their limitation to yield.

To allow trustful downscaling of nutrient diagnostic methods, regional diagnosis across factors must be coherent with diagnosis at a local scale where myriads of factor combinations occur. Growers solved this problem intuitively by conducting side-by-side comparisons between unhealthy and nearby healthy specimens. Compositional methods provide a quantitative compositional diagnostic approach by comparing defective to successful neighboring compositions at factor levels shared by the defective and successful specimens. Such side-by-side comparison also provides trustful attainable yields under the specified combination of factors. As shown by the discrepancy between regional and local diagnoses, the factor-specific approach could control errors attributable to factor dissimilarity potentially affecting crop yield at the local scale.

### 4.2. Compositions as Unique Combinations of Nutrients

Nutrient acquisition by plants depends on environmental factors such as soil properties, soil water content, and climatic conditions [52,53]. Nutrient combinations leading to high-yields under successful conditions at the specified factor levels may change as controllable growth-limiting factors are alleviated. While the Law of the Maximum relies on tens of growth factors and countless factor interactions [20], «Jardins do Eden», where all factors are at their optimum levels, are rarely encountered. On the other hand, «ilhas encantadas» (enchanting islands) [18,26,27], where controllable factors are close to their optima under given combinations of uncontrollable factors, can be documented as successful Humboldtian loci where several yield-limiting factors have been handled adequately by local growers.

At a local scale, under a given combination of uncontrollable and controlled factors shared by neighboring defective and successful specimens, assumptions on factors being equal or at optimum levels can be considerably reduced. Parent [26] depicted growers searching for maximum yield from a set of controllable growth-limiting factors as compositional parachutists trying to land on the nearest enchanting island by manipulating D-1 suspension lines at a time to avoid falling into the surrounding turbulent sea. Where low yield, DBH or plant vigor is observed and nutrient imbalance is suspected, the objective is to reach high nutrient-use efficiency by adopting reliable corrective measures already implemented in the successful neighborhood. To generate large, trustful, and informative data sets to conduct nutrient diagnoses at a local scale, a close and ethical collaboration is required between researchers and stakeholders [54].

## 5. Conclusions

The present Brazilian nutrient concentration ranges for Mg, Mn, Fe and Zn differed markedly from compatibility intervals derived from the TN specimens in the data set. Moreover, denying nutrient interactions, nutrient concentration ranges collapsed in the Euclidean space as more nutrients are added. Indeed, only one TN specimen survived after diagnosing 489 TN specimens across eleven nutrient compatibility intervals bounded by the TN quartiles. Although easy to interpret, dichotomous decisions inherited from the past using critical nutrient concentration ranges should be replaced by tools of machine learning and compositional data analysis.

The ML model showed that the productivity of young *Eucalyptus* trees depended not only on mineral nutrition but also on local features such as clone, soil type, location, and tree age. Raw concentrations returned higher model accuracy and were not affected by missing values compared to log-ratios. As a result, log-ratio transformations are solely required in data post-processing to integrate nutrient interactions in the diagnostic nutrient-ranking heuristic model.

Regional and local nutrient diagnoses of defective specimens may differ. As a result, downscaling regional nutrient standards to a local scale could be hazardous and could explain the large variation in fertilization regimes in Brazilian *Eucalyptus* ecosystems, where environmental and managerial factors vary widely. Local scale diagnosis by factor analogy is viable to reach potential yield levels. Factor-specific diagnosis has the advantage over regional diagnosis that local factors can be kept similar in every aspect but factors that have been controlled in the successful neighborhood.

Although the local diagnostic approach is appealing to avoid error on interactions, it is highly demanding in well-documented and trustful data. Meteorological data, pest management and soil quality tests could be further documented to increase *Eucalyptus* model accuracy. Commitment to share relevant information is essential to build large data sets and return accurate predictions. A close, trustful, and ethical collaboration is thus necessary between stakeholders to customize and validate tissue nutrient diagnosis of *Eucalyptus* trees at a local scale.

## Figures and Tables

**Table 1:** Ranges of tissue nutrient concentrations and filling values for 1861 young *Eucalyptus* trees in Southern Brazil.

Component	Minimum	Median	Maximum
	g kg^−1^		
N	9.1	21.9	38.8
P	0.5	1.3	3.3
K	1.2	9.4	19.6
Mg	1.0	2.6	7.5
Ca	2.7	8.6	34.9
S	0.4	1.5	5.1
B	0.011	0.038	0.105
Cu	0.001	0.008	0.036
Zn	0.006	0.018	0.129
Mn	0.066	0.964	4.954
Fe	0.002	0.076	0.594
Filling value	925.6	952.1	973.3

**Table 2:** Accuracies of ML classification models for the *Eucalyptus* data set (1861 observations) for nutrient concentrations and other features in cross-validation, using diameter at breast height (DBH) as the target variable at a BDH cut-off of 4.3 cm between high- and low-yielding trees.

Expression	AUC	CA	TN	FN	FP	TP
Random Forests	0.787	0.718	521	219	315	816
Neural Networks	0.778	0.705	548	271	278	764
Naïve Bayes	0.793	0.715	614	318	212	717
Support Vector Machine	0.544	0.529	-	-	-	-
KNN	0.589	0.570	-	-	-	-
Adaboost	0.636	0.641	-	-	-	-
Stochastic Gradient Decent	0.674	0.679	-	-	-	-

AUC = area under the curve (≥0.7 required); CA = classification accuracy; TN = true negative; FN = false negative; FP = false positive; TP = true positive.

**Table 3:** Comparison of accuracies from nutrient expressions using the RF model to process the Eucalyptus tree data set (1861 observations) in cross-validation.

Nutrient Expression	Area Under Curve	Classification Accuracy
Raw concentration data	0.787	0.718
Pairwise log ratios	0.721	0.664
Centered log ratios	0.785	0.706
Isometric log ratios	0.776	0.701

**Table 4:** State concentration ranges and quartile compatibility intervals (0.25, 0.75) nutrient values of 489 TN specimens of 0.9–1.1-year-old *Eucalyptus*.

Nutrient	State (Gatiboni et al. [11])		True Negative Quartiles (25, 75)	
	Lower bound	Upper bound	Lower bound	Upper bound
	g kg^−1^			
N	15.0	20.0	17.0	25.3
P	1.0	1.3	1.0	1.4
K	9.0	13.0	7.2	11.5
Mg	6.0	10.0	2.3	3.2
Ca	5.0	8.0	7.0	10.2
S	1.5	2.0	1.2	1.8
	mg kg^−1^			
B	30	50	6	12
Cu	7	10	14	21
Zn	35	50	60	96
Mn	400	600	34	54
Fe	150	200	679	1281

**Table 5:** Nutrient concentrations of fictive specimens diagnosed by state standards and TN quartiles in Table 4 (italicized diagnoses are similar).

Nutrient	Site #1	Site #2	Site #1		Site #2	
	g kg^−1^		State Standards	TN Quartiles	State Standards	TN Quartiles
N	27.1	15.0	*High*	*High*	Normal	Low
P	1.4	1.3	High	Normal	*Normal*	*Normal*
K	8.8	8.2	Low	Normal	Low	Normal
Mg	1.5	3.8	*Low*	*Low*	Low	High
Ca	3.9	21.2	*Low*	*Low*	*High*	*High*
S	1.7	1.4	*Normal*	*Normal*	Low	Normal
	mg kg^−1^		Diagnosis			
B	48.0	1.3	Normal	High	*Low*	*Low*
Cu	4.7	17.9	*Low*	*Low*	High	Normal
Zn	14.7	151.8	*Low*	*Low*	*High*	*High*
Mn	452.3	73.8	Normal	High	Low	High
Fe	66.9	1614.4	*Low*	*Low*	*High*	*High*

**Table 6:** Centered log ratio statistics for 0.9–1.1-year-old TN specimens of *Eucalyptus* as regional nutrient standards across features.

Nutrient	489 TN Specimens	
	**Mean**	**Standard Deviation**
N	2.9050	0.3048
P	0.0726	0.2618
K	2.1387	0.2878
Mg	0.8880	0.2270
Ca	2.0454	0.2962
B	−4.8438	0.4189
S	0.3053	0.3024
Cu	−4.0882	0.3872
Zn	−2.7212	0.4089
Mn	−3.2629	0.3338
Fe	−0.2132	0.4754
Fv	6.7743	0.1453
